# Chromatin Structure of Ribosomal RNA Genes in Dipterans and Its Relationship to the Location of Nucleolar Organizers

**DOI:** 10.1371/journal.pone.0044006

**Published:** 2012-08-30

**Authors:** Christiane Rodriguez Gutierrez Madalena, José Luís Díez, Eduardo Gorab

**Affiliations:** 1 Departamento de Genética e Biologia Evolutiva, Instituto de Biociências, Universidade de São Paulo, São Paulo, Brazil; 2 Departamento de Biología Celular y del Desarrollo, Centro de Investigaciones Biológicas, Consejo Superior de Investigaciones Científicas, Madrid, Spain; St. Georges University of London, United Kingdom

## Abstract

Nucleoli, nuclear organelles in which ribosomal RNA is synthesized and processed, emerge from nucleolar organizers (NORs) located in distinct chromosomal regions. In polytene nuclei of dipterans, nucleoli of some species can be observed under light microscopy exhibiting distinctive morphology: *Drosophila* and chironomid species display well-formed nucleoli in contrast to the fragmented and dispersed nucleoli seen in sciarid flies. The available data show no apparent relationship between nucleolar morphology and location of NORs in Diptera. The regulation of rRNA transcription involves controlling both the transcription rate per gene as well as the proportion of rRNA genes adopting a proper chromatin structure for transcription, since active and inactive rRNA gene copies coexist in NORs. Transcription units organized in nucleosomes and those lacking canonical nucleosomes can be analyzed by the method termed psoralen gel retarding assay (PGRA), allowing inferences on the ratio of active to inactive rRNA gene copies. In this work, possible connections between chromosomal location of NORs and proportion of active rRNA genes were studied in *Drosophila melanogaster,* and in chironomid and sciarid species. The data suggested a link between location of NORs and proportion of active rRNA genes since the copy number showing nucleosomal organization predominates when NORs are located in the pericentric heterochromatin. The results presented in this work are in agreement with previous data on the chromatin structure of rRNA genes from distantly related eukaryotes, as assessed by the PGRA.

## Introduction

Ribosomal RNA (rRNA) represents the most abundant transcription product in prokaryotes and eukaryotes and together with proteins forms the ribosome. Ribosome biogenesis starts in the nucleolus, the nuclear territory where rRNA is transcribed and processed. Genes encoding rRNA (rDNA) are nucleolar components genomically structured as tandem repeats of variable number and their activity define the nucleolar organizer region (NOR) which can be present in one or more chromosomes.

Under light microscopy, polytene nuclei of Diptera allow a magnified view of nuclear compartments, the details of which cannot be achieved in diploid cells. Polytene nucleoli may thus be observed displaying distinct morphology depending on the species studied. For example, *Chironomus* (Suborder Nematocera) and *Drosophila* (Suborder Brachycera) species are distantly related dipterans that usually exhibit well-formed nucleoli [1], [2]. In contrast, nucleoli in sciarid species (Sub-Order Nematocera) show an irregular form in polytene cells and tend to fragment and disperse throughout the larval development. Micronucleoli are also nucleolar material in sciarids and appear as round bodies scattered in the nucleoplasm, frequently associated with certain chromosome regions [3]–[5].

In *Drosophila*, NORs are localized in the heterochromatin of the X and Y chromosomes [6]. Sciarid species usually display, as in *Drosophila*, NORs at the pericentric heterochromatin of the X chromosome [7], [8]. However, NORs in some chironomid species are not localized in the heterochromatin. As examples, *Camptochironomus tentans* (formerly named *Chironomus tentans*) has two NORs localized interstitial sites of chromosomes II and III [1] while *Chironomus riparius* (formely named *C. thummi*) has a single NOR within a region in chromosome IV that does not coincide with the centromere or telomeres [9].

Control of the rRNA gene activity is critical for cell function and it is likely to operate concertedly at three levels. The transcription rate can be controlled either at the RNA polymerase I (Pol.I) promoter or by modulating the elongation of Pol.I [10]. The third level is determined by the establishment of a proper ratio between transcriptionally active and silenced rDNA, an epigenetic process that takes place in the course of cell differentiation. In relation to the latter, except for rare cases, a significant fraction of the rDNA is not transcribed in eukaryotic nuclei and thus active and inactive rRNA gene copies coexist in NORs. It has been demonstrated that transcriptionally active rDNA units are devoid of canonical nucleosomes and are more accessible to psoralen crosslinking than the silent units that exhibit nucleosomal organization. Differential electrophoretic migration of the DNA from chromatin populations that underwent psoralen crosslinking allows the separation and quantification of active and inactive rRNA gene copies after Southern-blot hybridization. Psoralen gel retarding assay (PGRA) has been proven to be the most sensitive method for identifying chromatin structures of the rDNA and their relationship to transcription [11].

With regard to dipterans, the above technique was employed for the first time in embryonic cells of *D. melanogaster* and results suggested a very low proportion (<10%) of rDNA engaged in transcription since the hybridization band corresponding to the active copies could not be detected by the PGRA [12]. On the other hand, use of the same method in *C. riparius* cells showed measurable, significantly different tissue-dependent proportions of rDNA free from nucleosomes and representing transcriptionally active rDNA chromatin [13].

From the data described above, it is clear that the distinct nucleolar morphology of sciarid flies compared to that of *Drosophila* and *Chironomus* is not related to the chromosomal localization of NORs. The questions raised in this work are concerned with possible links between nucleolar variables in the salivary gland of dipterans such as proportion of transcriptionally active rDNA copies and chromosomal location of NORs. In order to confirm whether and how these variables are connected, the PGRA was applied to study the chromatin structure of rRNA genes from three sciarid species, *Drosophila melanogaster* and *Camptochironomus tentans*. These organisms display NORs in either euchromatic or heterochromatic regions as specified above. In addition, they can be kept as laboratory cultures in contrast to many other dipteran species.

The data obtained suggested that the chromosomal location of NORs in the pericentric heterochromatin seemed to have a significant impact on the chromatin structure of rDNA repeats. This was inferred by the very low copy number of rRNA genes engaged in transcription in the salivary gland as well as in other cell types of *Drosophila* and sciarid species. Such an assumption, made on the basis of results obtained in this work, is additionally supported by data from other eukaryotes whose rDNA chromatin was also analyzed by the PGRA.

## Results

### Psoralen Crosslinking Pattern in the Ribosomal Chromatin of Sciarid Flies

**Figure 1:**
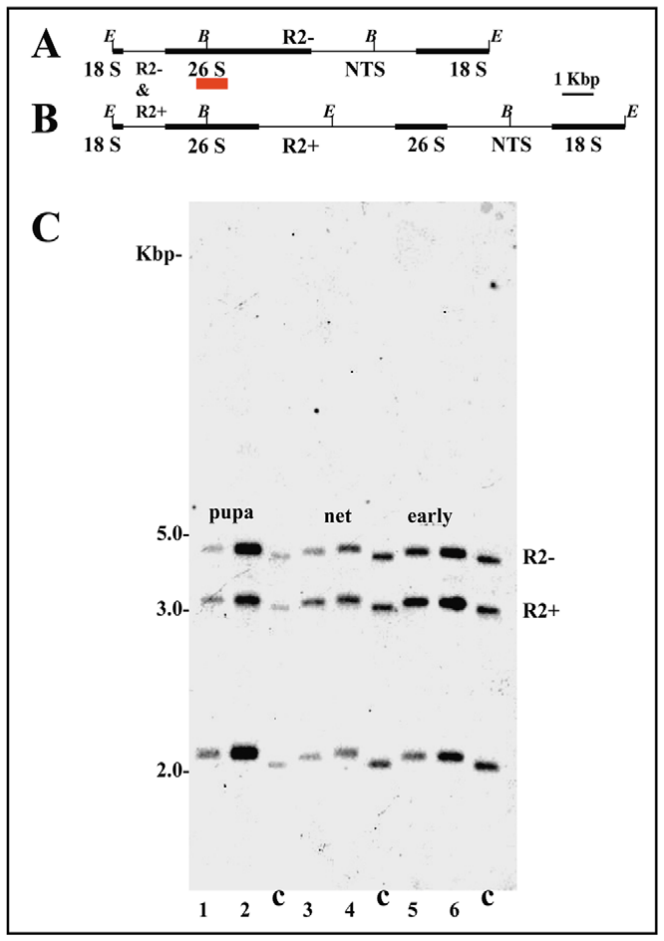
Chromatin structure of rRNA genes in the salivary gland of *Rhynchosciara americana*. (A, B) rDNA maps of *R. americana*, modified from [14], showing *Eco*RI (*E*) and *Bgl*I (*B*) restriction sites in uninterrupted (A) and R2-containing (B) rDNA repeats. Position of the probe [47] relative to the rDNA repeat unit is indicated with a red bar; hybridization bands specific for canonical rDNA units (R2−) and those interrupted by R2 insertions (R2+) are identified. (C) Southern-blot hybridization analysis of rDNA fragments obtained by digesting genomic DNA with *Eco*RI/*BglI* from control DNA (c) and from salivary gland chromatin photo-reacted in the presence of psoralen (1–6). Samples numbered 1, 3 and 5 came from female larvae, even samples came from male larvae. DNA from larvae still feeding (eat), in the beginning of cocoon construction (net) and close to the head eversion stage (pupa) were used in the experiments.

According to previous results obtained with this species [14], the rDNA probe of *Chironomus* should hybridize to three *Eco*RI/*Bgl*I restriction fragments in Southern-blots of *Rhynchosciara americana* DNA (Figure 1A, B). The largest *Bgl*I restriction band (approximately 4.3 kbp) represents rDNA repeat units without R2 elements (R2−). A second *Eco*RI*/Bgl*I fragment (approximately 3.4 kbp) corresponds to rDNA repeats with R2 insertions (R2+). The third band (around 2.3 kbp) contains a mixture of restriction fragments from both rDNA repeat types (R2− and R2+). The results shown were representative of five Southern-blot experiments in which DNA samples from distinct larval groups were examined. The only rDNA fragment detected by the PGRA corresponds to that of nucleosomal rRNA gene copies. The rDNA chromatin structure was found to be the same when restriction fragments of rDNA units containing R2 insertions and those free from insertions were compared.

**Figure 2:**
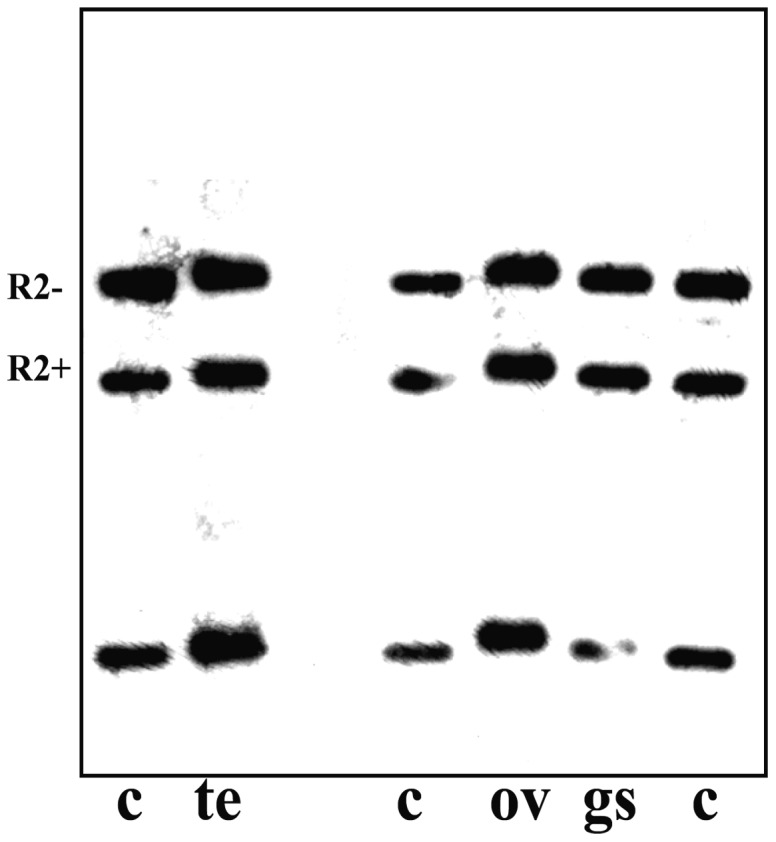
Chromatin structure of rRNA genes in the salivary gland, ovary and testis of *Rhynchosciara americana*. Southern-blot hybridization analysis of rDNA fragments obtained by digesting with *Eco*RI/*BglI* genomic DNA from salivary gland (sg), ovary (ov) and testis chromatin (te) photo-reacted in the presence of psoralen and also from control, untreated DNA (c). Hybridization bands specific for canonical rDNA units (R2−) and those interrupted by R2 insertions (R2+) are indicated.

A significant decrease in the rRNA synthesis was detected after the highest ecdysone peak that signals late prepupal instar of *R. americana* [15]. This observation could be related to changes in the chromatin structure of ribosomal genes affecting the proportion of active rDNA units. However, the results showed no apparent change in the proportion of transcriptionally active ribosomal chromatin when either male or female larvae from distinct developmental periods were used in the experiments (Figure 1C). DNA from the salivary gland chromatin of *Chironomus riparius* was used as a control and two bands representing transcriptionally active and inactive rDNA copies were invariably detected. The results obtained with ribosomal chromatin from the salivary gland of *R. americana* raised the question of whether they are a feature restricted to polytene tissues. The PGRA was then applied to the chromatin from ovaries and testes and the results were the same as those observed in the salivary gland (Figure 2).

**Figure 3:**
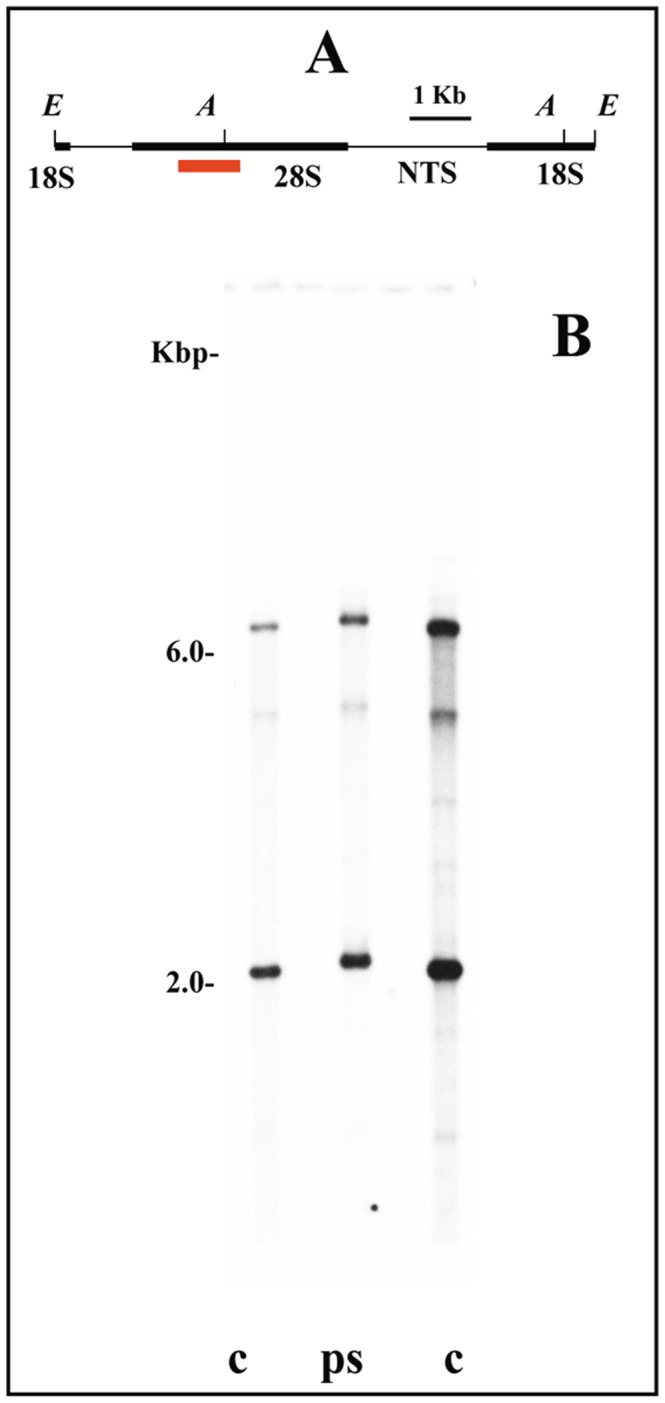
Chromatin structure of rRNA genes in the salivary gland of *Sciara coprophila*. (A) rDNA map of *S. coprophila*, modified from [48] showing (red bar) the position of the probe [47] relative to the rDNA repeat unit. *Eco*RI (*E*) and *Ava*I (*A*) restriction sites are indicated in the map. (B) Southern-blot hybridization analysis of rDNA fragments obtained by digesting genomic DNA with *Eco*RI/*Ava*I from salivary gland chromatin photo-reacted in the presence of psoralen (ps) and from control, untreated DNA (c).

Like most sciarid species studied so far, *Sciara coprophila* has its rDNA cluster localized in the proximal heterochromatin of the X chromosome. In contrast to the results observed in *C. riparius* [13] and in agreement with those obtained in *R. americana*, bimodal distribution of restriction fragments from the rDNA chromatin of *S. coprophila* was not observed after many attempts. A single band migrating as nucleosomal rDNA was detected even when massive amounts of DNA from the chromatin were used in Southern-blots (Figure 3B). The same results were obtained when larvae from distinct developmental periods were used in the experiments. Again, controls done in parallel with chromatin DNA from the salivary gland of *C. riparius*, using the same equipments and reagents, showed two rDNA bands representing two different chromatin structures as demonstrated previously [13]. The controls thus allowed us to rule out any technical problem in the assays performed with this species. Faint hybridization bands are not partial DNA digestion products but corresponded to ribosomal RNA gene insertion variants characterized in this species [16]. The results shown in this work were representative of nine Southern-blot experiments performed.

**Figure 4:**
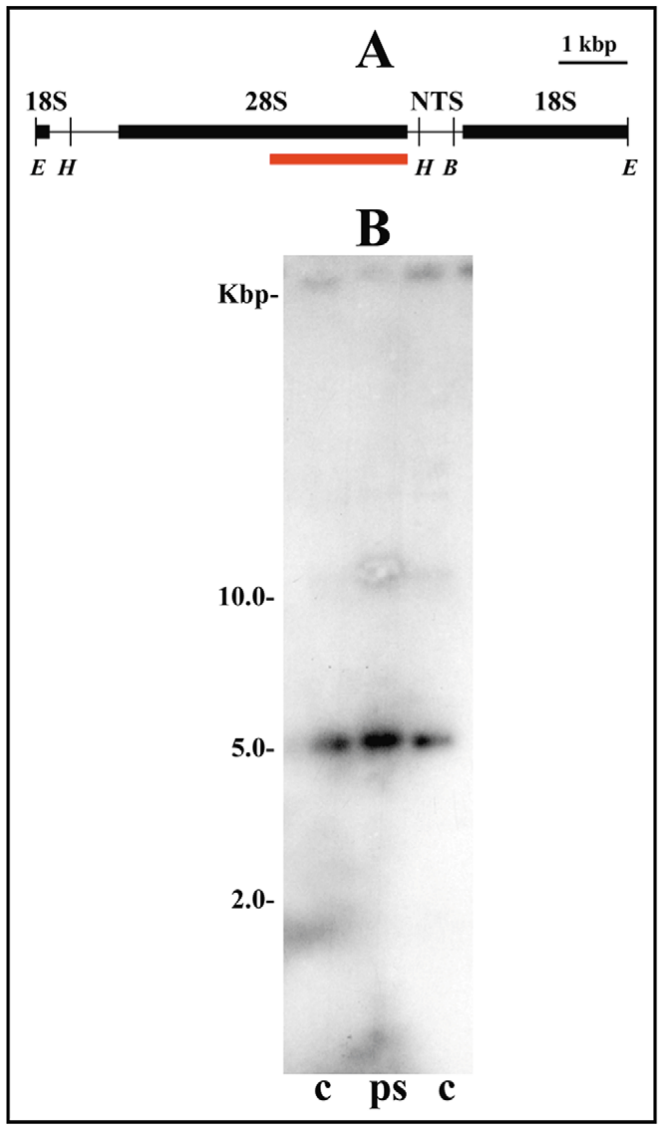
Chromatin structure of rRNA genes in the salivary gland of *Trichomegalosphys pubescens*. (A) rDNA map of *T. pubescens* showing (red bar) the position of the 26 S rDNA probe [14] used for hybridizations. Positions of *Eco*RI (*E*), *Hind*III (*H*) and *Bgl*I (*B*) restriction sites are indicated. (B) Southern-blot hybridization analysis of rDNA fragments obtained by *Hind*III digestions of genomic DNA from salivary gland chromatin photo-reacted in the presence of psoralen (ps) and from control, untreated DNA (c).

The rDNA cluster in *Trichomegalosphys pubescens* is localized in the pericentric heterochromatin of the chromosome X, sections X8–X10 [8]. Studies on the characterization of the heterochromatin and rDNA underreplication in polytene nuclei have not been carried out yet in this sciarid species. It is known that polytene chromosome sections comprising X8–X10 have no well-defined banding pattern and contain the chromosome X breakpoint, suggesting local DNA underreplication. In the course of the experiments for the rDNA map construction, DNA restriction bands representing rDNA retrotransposons were not detected, in agreement with a previous study [14]. The results on the chromatin structure of ribosomal RNA genes in the salivary gland of this species were the same as those observed in other sciarid species, namely hybridization bands representing transcriptionally active rDNA copies could not be detected after several attempts (Figure 4B). Also, no change in the ribosomal chromatin structure was detected when either male or female larvae from distinct developmental periods were used in the experiments or even when the chromatin of ovaries and testes were examined (data not shown).

### Proportions of Open rDNA Chromatin in *Camptochironomus Tentans* and *Drosophila*

**Figure 5:**
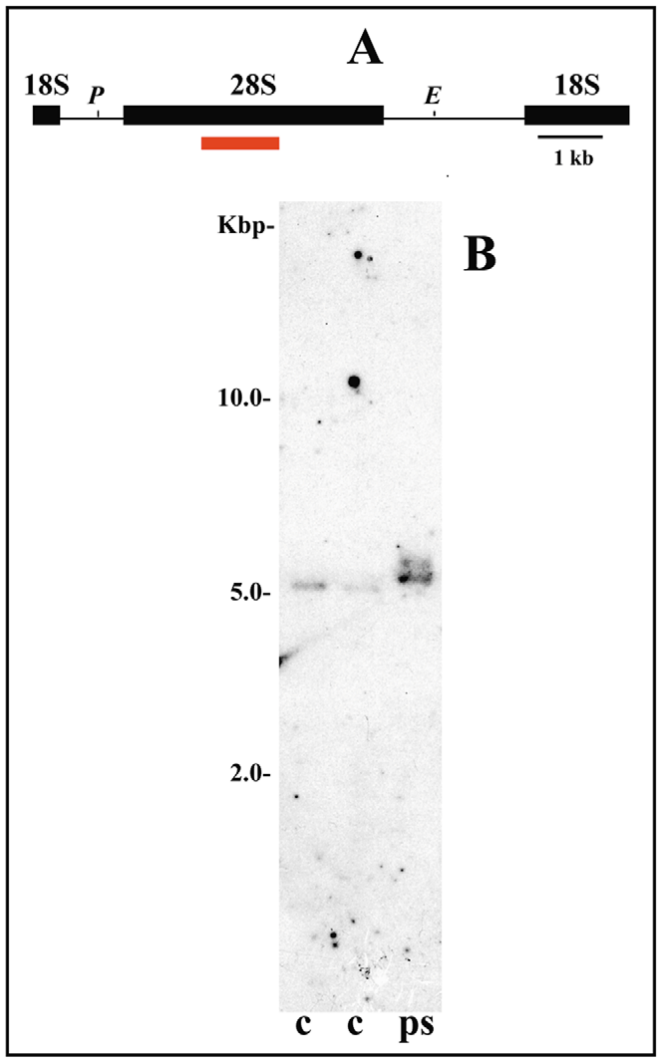
Chromatin structure of rRNA genes in the salivary gland of *Camptochironomus tentans*. (A) rDNA map of *C. tentans*, modified from [17], showing (red bar) the position of the probe [15] relative to the *C. tentans* rDNA repeat unit. *Eco*RI (*E*) and *Pst*I (*P*) restriction sites are indicated in the map. (B) Southern-blot hybridization analysis of the 28S rDNA fragments obtained by digesting with *Eco*RI/*Pst*I genomic DNA from salivary gland chromatin photo-reacted in the presence of psoralen (ps) and from control, untreated DNA (c).

In relation to Chironomidae, the chromatin structure of rRNA genes in larval tissues of *Chironomus riparius* was studied using PGRA [13]. The results showed a bimodal distribution of restriction fragments corresponding to active and inactive rDNA repeat units that are clustered in chromosome IV. In this work, the rDNA chromatin of another chironomid species, *Camptochironomus tentans*, was assessed by the same technique. This species displays two nucleolar organizers that, like *C. riparius*, are not localized in the pericentric heterochromatin. Restriction fragments corresponding to ribosomal retrotransposons were not characterized in this species [17] and were not detected after several Southern-blot hybridizations performed in this work. As observed in *C. riparius*, PGRA results showed that a given rDNA restriction fragment from the chromatin of *C. tentans* migrates as two bands of distinct mobility, representing nucleosomal and non-nucleosomal rDNA copies (Figure 5B). Densitometric values from three experiments suggest that 40% (±3% SE) of the rDNA copies are structured as transcriptionally active chromatin in the salivary gland of this species.

**Figure 6:**
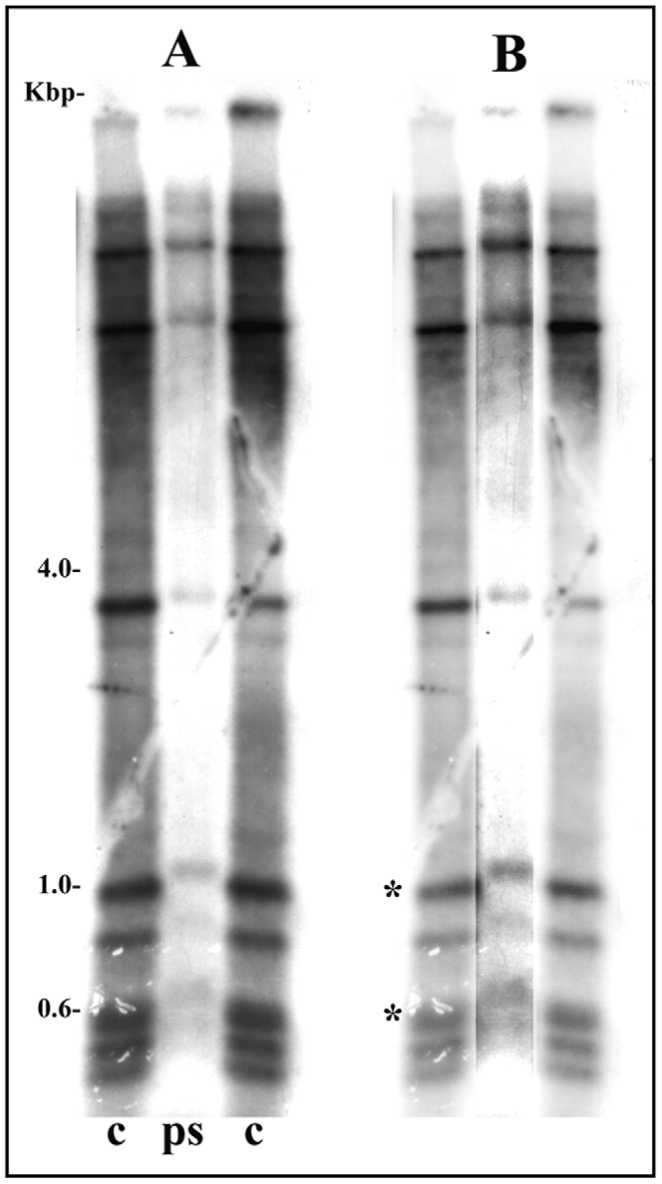
Chromatin structure of rRNA genes in the salivary gland of *D. melanogaster*. (A) Southern-blot hybridization analysis of rDNA fragments obtained by *Hae*III digestions of genomic DNA from salivary gland chromatin photo-reacted in the presence of psoralen (ps) and from control, untreated DNA (c). The whole insert of the p*Dm*-238 [18] was used as a probe. (B) Each lane from the same image shown on the left was processed for brightness, contrast, and signal intensity in order to improve visualization of radioactive signals. The asterisks indicate the two bands detected when Southern-blot hybridization using the same membrane was performed with the *Chironomus* 26S rDNA probe [47].

The results presented up to this time have shown that the distinct nucleolar morphology displayed by sciarid and chironomid species is associated with significantly different proportions of rDNA copies devoid of nucleosomal structure. A third nucleolar variable in these species is the location of NORs. Information on the chromatin structure of *Drosophila melanogaster* rDNA is important for the study of relationships between these variables since nucleolar morphology in *Drosophila* resembles that of Chironomidae while location of *Drosophila* NORs is pericentric as in sciarid species. Previous data from *D. melanogaster* embryos have suggested a very low proportion of transcriptionally active rDNA repeats [12] but the chromatin structure of rRNA genes in the salivary gland of this species has not yet been assessed by PGRA. The whole rDNA repeat unit of *D. melanogaster*, p*Dm*-238 [18], was first used as a probe in Southern-blot hybridization of control and psoralen-treated DNA cut with *Hae*III. As expected, several restriction fragments hybridized to the probe and, in agreement with data from photoreacted embryonic chromatin [12], bands representing non-nucleosomal rDNA were not detected in salivary glands. The 26S rDNA probe of *C. riparius* hybridized to two *Hae*III bands migrating at approximately 1.0 and 0.6 kbp (Figure 6).

**Figure 7:**
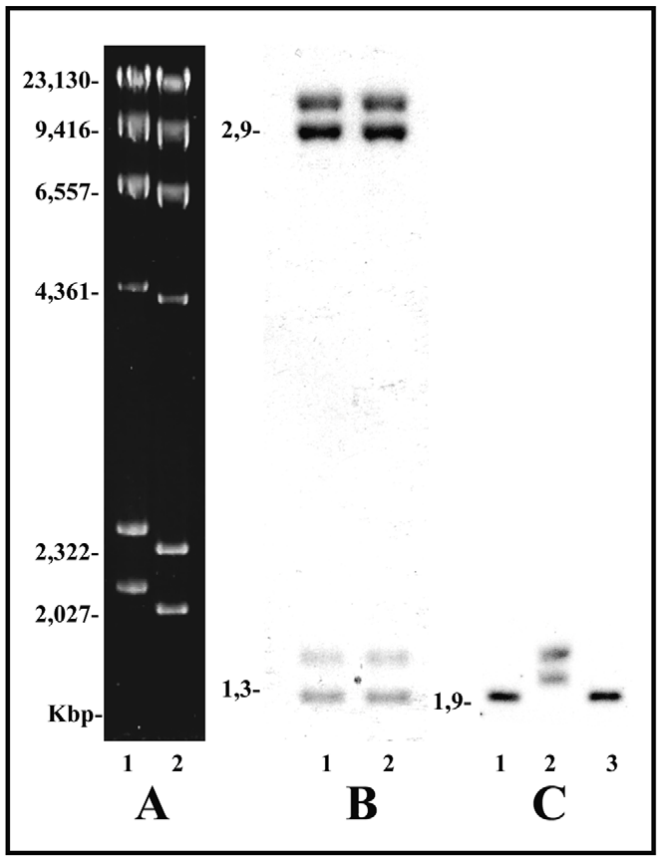
PGRA applied to naked and chromatin DNA. (A) Lambda DNA cut with *Hind*III was psoralen crosslinked and analyzed in agarose gel (1.2%) for 20 h at 44V (lane 1) together with non-crosslinked Lambda DNA (lane 2). (B) DNA fragments of definite size were photoreacted in the presence of psoralen and then mixed with non-crosslinked DNA fragments of the same length. The mixture was loaded into two lanes (1,2) and run in agarose gel (1.2%) for 14 h at 60V. Nucleic acids were transferred and hybridized to the probe synthesized from non-crosslinked DNA fragments. (C) Salivary gland cells of *Chironomus riparius* were photoreacted in the presence of psoralen, following DNA extraction, digestion with *Hae*III and electrophorectical analysis in agarose gel (1.2%) at 60V for 17 h. DNA in the gel was transferred and hybridized to the 26S rDNA probe (lane 2). Untreated genomic DNA used as a control was analyzed in parallel (lanes 1, 3).

Except for salivary gland cells of *C. tentans* and *C. riparius*, ribosomal genes from the sciarid flies and *Drosophila* that have been analyzed by the PGRA in this work appeared as a single chromatin conformation. This band has a slightly reduced mobility when compared with control DNA implying nucleosomal configuration. The conclusion is also supported by controls in which purified DNA fragments of definite size are psoralen-treated and analyzed in gels together with untreated DNA. The migration behavior of naked DNA after crosslinking could be viewed as a non-nucleosomal DNA marker. Photoreaction results obtained with naked DNA (Figure 7A, B) showed that, under electrophoretic conditions employed in most experiments, crosslinked and non-crosslinked fragments of the same length can be readily separated within a size range (1,3–5,5 Kbp) comprising the majority of rDNA bands analyzed this work. When the PGRA results showed chromatin resolved into a single rDNA band, its migration is always closer to that expected for non-crosslinked DNA. In contrast, bands representing non-nucleosomal DNA as seen with either chromatin rDNA or naked DNA after crosslinking display significantly reduced mobility (Figure 7B, C).

### H3K9 Methylation Levels at rDNA Loci of *Drosophila* and Sciarid Flies

Antibodies to histone post-translational modifications have shown that methylation of histone H3 at lysine 9 (H3K9) are among the predominant marks of pericentric heterochromatin in *Drosophila* [19]. In addition, chromatin immunoprecipitation suggested that dimethylation of H3K9 has a role in silencing both inserted and uninserted rDNA units of *Drosophila* [12] and that mutations in genes encoding histone methyltransferases disturb the nucleolar structure [20]. In *Sciara coprophila*, the rDNA locus located in the pericentric heterochromatin of the X chromosome displays a significant enrichment with chromodomain-containing proteins and also with di- and trimethylation of H3K9 [21] as seen in *Drosophila*. Interaction of these two molecular components leads to the formation of silencing complexes localized in the pericentric heterochromatin [22] which are probably connected to published results on the chromatin structure of rRNA genes in *Drosophila* [12] and also those obtained in this work. Given that a similar picture also seems to occur in *S. coprophila*, as suggested by previous data [21] and our PGRA results, such a possibility might be strengthened if immunocytochemical data from *R. americana* and *T. pubescens* were provided. Detection of trimethylation of H3K9 was then carried out for the first time in chromosomes of these two sciarid species.

**Figure 8:**
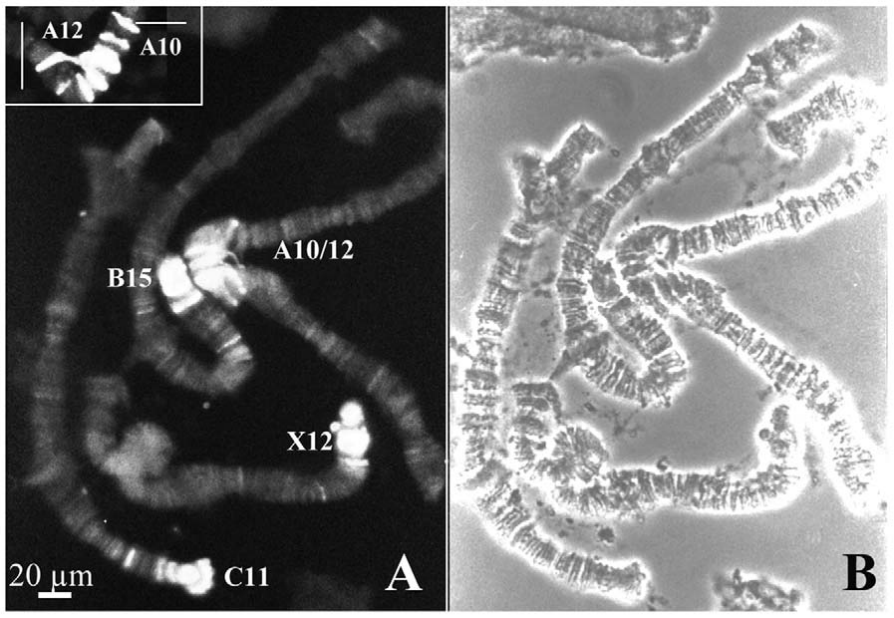
Chromosomal distribution of trimethyl H3K9 in *R. americana*. (A) Indirect immunofluorescence; the insert shows labeling details of chromosome sections A10/12 comprising pericentric heterochromatin of chromosome A. (B) The corresponding phase contrast image. Polytene chromosome sections were identified according to a previous report [8].

**Figure 9:**
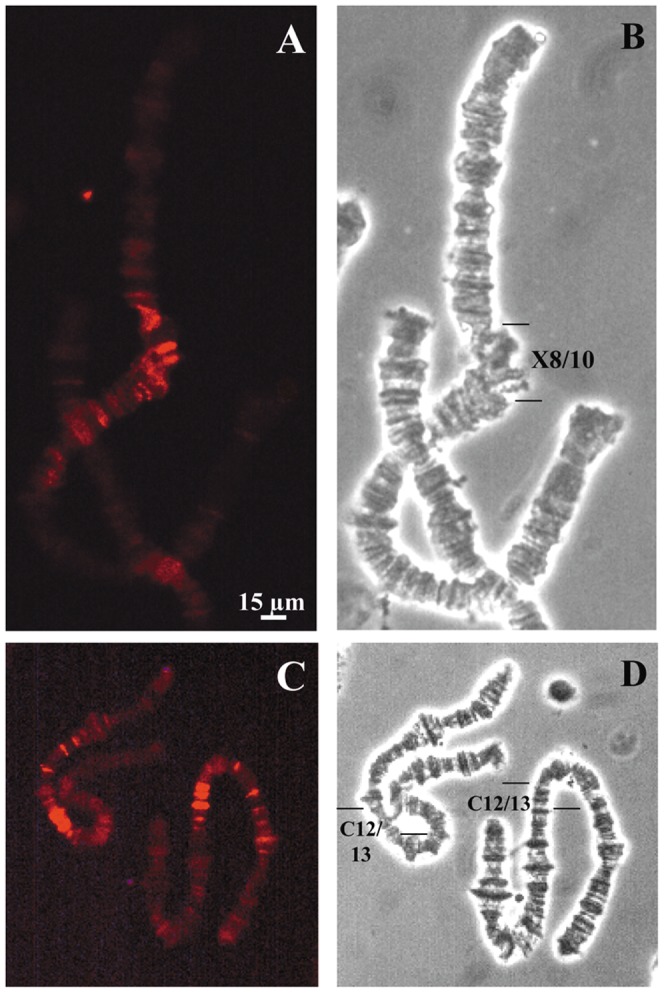
Chromosomal distribution of trimethyl H3K9 in *T. pubescens.* (A) Indirect immunofluorescence. (B) The corresponding phase contrast image; bars indicate the limits of chromosome X sections identified in a previous study [8]. (C) Indirect immunofluorescence showing staining in two chromosomes C. (D) The corresponding phase contrast image; bars point the limits of pericentric chromosome sections containing the strongest signals.

In *R. americana*, pericentric regions comprising chromosome sections B15, C11, X12 and A10/12 were strongly stained by the antibody (Figure 8). In polytene chromosomes of this species, rDNA probes hybridized to pericentric sections B15, C11 and X12 [8] that overlap clearly chromatin areas enriched with trimethylation of H3K9. In the chromosome X of *T. pubescens*, sections X8/10 appeared significantly labeled by the antibody (Figure 9A), remarkably coinciding with the rDNA location in this species [8]. Strong detection of H3K9 trimethylation in the polytene complement of *T. pubescens* overlaps pericentric regions mapped previously (example in Figure 9C).

## Discussion

Five genera representing three dipteran families were exploited in this work in order to study the chromatin structure of rRNA genes in the salivary gland and in other larval tissues as visualized by the PGRA. The choice of this method is mainly related to its accuracy in measuring percentages of non-nucleosomal rDNA copies representative of transcriptionally active rRNA genes as shown in a number of previous reports. Except for *C. tentans*, the overall results showed that the proportion of rDNA repeat units adopting non-nucleosomal configuration could not be detected by the PGRA. Thus, the copy number of transcribing rDNA in the salivary gland of *D. melanogaste*r and sciarid species studied in this work is deduced to be below the limit of PGRA detection, which means values lower than 10%.

PGRA applied to embryonic cells of *D. melanogaster* allowed the postulation that approximately 24 rDNA repeats are engaged in transcription [12]. In the salivary gland of *Drosophila*, underreplication leads to approximately 10-fold reduction in the number of rRNA genes [23]. In *R. americana*, ovary cells have around 220 rRNA genes per haploid genome while rDNA repeats in the salivary gland are reduced to 100 units as a consequence of underreplication [24]. By comparing the data from the two species, rDNA repeat number is higher in the salivary gland of *R. americana*. Transcriptionally active rRNA genes in each species are likely to be proportional to the total amount of rDNA repeats in the salivary gland. From this statement, one may conclude that the distinct nucleolar morphology observed in *Rhynchosciara* and other sciarid species when compared to *Drosophila* does not seem to be related to the number of rDNA units engaged in transcription. Experiments in which a single rRNA gene copy was inserted in the euchromatin of *Drosophila* demonstrated that a nucleolus was formed at the rDNA insertion site [25]. Therefore, a well-formed nucleolar structure can be generated from a single, transcriptionally active rDNA repeat.

DNA underrepresentation in the heterochromatin has long been characterized as a feature of polytene tissues. Except for *T. pubescens*, underreplication of rDNA in the salivary gland has already been demonstrated in *Drosophila* [23], [26], [27], *Sciara coprophila* [28] and in *R. americana* [24]. Nevertheless, rDNA underreplication was not detected in the salivary gland of *C. tentans* [29], as expected from its localization out of the heterochromatin according to previous studies [1], [17], [29]. Data obtained in *Drosophila* and sciarid species link underreplication, a mark of heterochromatinization in polytene tissues, and rDNA mostly structured as nucleosomes. Distinct chromosomal compartments may thus contribute differentially to the number of rDNA copies engaged in transcription.

The low proportion of transcriptionally active rDNA in *Drosophila* was first suggested from ultrastructural data [30]–[32]. PGRA results showed the chromatin structure of the rDNA predominantly nucleosomal in embryonic cells of *D. melanogaster*, irrespective of the insertion of R1/R2 elements [12]. Use of the same method in this work extended the above observations to the *Drosophila* salivary gland and also ribosomal chromatin of *R. americana*. The rDNA units in this species probably have the highest frequency of insertion into the R2 site when compared to *S. coprophila* and *T. pubescens* [14], [16]. Consistent with the data obtained in *Drosophila*, the chromatin structure of rRNA genes in *R. americana* seems to be independent of rDNA insertions in all the tissues analyzed.

The reduced number of rDNA repeats adopting non-nucleosomal configuration might impose limits for the control of ribosome biogenesis. Such a constraint could be overcome at the transcriptional level by increasing Pol.I density and/or Pol.I elongation. On the other hand, it might be hypothesized that localization of NORs in pericentric heterochromatin dispense DNA methylation, intergenic RNAs and specific chromatin remodeling complexes that are responsible for silencing vertebrate rRNA genes [10]. Although negative evidence does not constitute proof, such processes and components have not been characterized yet in the rDNA chromatin of *Drosophila*.

More recently, use of immunoprecipitation for assessing the chromatin structure of yeast rRNA genes led to conclude that transcription proceeds throughout nucleosomal rDNA [33]. PGRA applied to the same yeast strain suggests that transcriptionally active rDNA is rather devoid of canonical nucleosomes [34], in agreement with all the data reported previously using the same method (Table 1). It remains conjectural at present whether those observations made on the basis of immunoprecipitation can be extended to *Drosophila* and sciarid species.

**Table 1:** Chromosomal location and proportion of active rRNA genes in eukaryotes.

Organism	Localization of the rRNA genes	Percentage of active rRNA genes
*Camptochironomus tentans*	Non-pericentric [1], [17], [29]	40 (x)
*Chironomus riparius*	Non-pericentric [9]	20–80 [13]
*Drosophila melanogaster*	Pericentric [6]	very low [12], (x)
*Lycopersicon esculentum*	Pericentric [35]	very low [36]
*Mus musculus*	Non-pericentric [37]	50 [38]
*Rattus norvegicus*	Non-pericentri [37]	30–35 [39]
*Rhynchosciara americana*	Pericentric [8]	very low (x)
*Saccharomyces cerevisiae*	Non-pericentric [40]	40–50 [34], [41], [42]
*Trichomegalosphys pubescens*	Pericentric [13]	very low (x)
*Xenopus borealis*	Non-pericentric [43]	50 [44]
*Xenopus laevis*	Non-pericentric [45], [46]	50 [44]

Proportion of active rRNA genes as inferred by the PGRA together with the respective chromosomal location of NORs in eukaryotes named in the first column. Values defined as very low mean that rDNA copies devoid of canonical nucleosomes are below 10%. Data obtained in this work were indicated (x).

The chromatin structure of the rDNA in distantly related eukaryotes, from yeast to mammals, has been studied by the PGRA. Secondary constrictions are signatures of NORs in vertebrate chromosomes and their occurrence indicates a particular chromatin structure in which the “Upstream Binding Factor” (UBF) seems to play an important role [10]. Connected to the latter, nucleosomal and non-nucleosomal rDNA were detected in mammals and amphibians in the form of two discrete bands (Table 1) implying a measurable proportion of rDNA repeats available for transcription. Interestingly, NORs in those organisms are not in chromosomal regions defined as centromeric, pericentric, intercalary or telomeric heterochromatin. Methylation patterns of H3K9 have been found in the rDNA chromatin of these species, more specifically in promoter regions of inactive genes [10]. To our knowledge however, such histone modifications have never been characterized as a preponderant mark either in secondary constrictions or in the yeast NOR, in which the bimodal pattern of the rDNA chromatin is usually visualized by the PGRA.

In contrast to vertebrates and yeast, rDNA repeats of *D. melanogaster* and sciarid species are predominantly structured as canonical nucleosomes and are located in the pericentric heterochromatin. Significant levels of methylation of H3K9 are a conserved feature of this chromosome region not only in the dipterans mentioned above but also in many species and could justify the PGRA results obtained in this work. Also, location of *Lycopersicon* rRNA genes in pericentric, heterochromatic knobs (Table 1), perhaps not by chance, correlates with its rDNA chromatin resembling that of *Drosophila* and sciarid species. Although epigenetic marks of *Lycopersicon* heterochromatin have not been described yet, the chromatin structure of tomato rRNA genes possibly undergoes the effects of similar silencing complexes, in which methylation of H3K9 and chromodomain-containing proteins take part, that are of widespread occurrence in pericentric regions if compared to other chromosome territories.

The phenomenon termed position effect variegation (PEV), discovered in *Drosophila* [50] has established a link between heterochromatin location and gene silencing. PGRA results obtained with rDNA arrays localized in the heterochromatin might be an expected manifestation of PEV. However, the heterochromatic localization of ribosomal and many other genes [51] would appear to be a contradiction in genome evolution. Why have such genes been evolutionarily maintained in a position that is repressive for their transcription? An explanation for this may be found in results showing that a fraction of the rRNA genes detected in all eukaryotic cells is inactive. It is possible that such inactivation is required for normal cell function and, in this case, the location of the rDNA repeats in heterochromatin where they are subject to PVE would be an efficient silencing mechanism. In the absence of PEV, other, and perhaps more complicated silencing mechanisms would be necessary.

In summary, the data obtained in this work using PGRA suggest that the low proportion of rRNA genes in *D. melanogaster* as well as in sciarid species, as visualized by their essentially nucleosomal structure, is related to their location in the pericentric environment. Such an assumption can be extended to other eukaryotes according to previous studies on the chromatin structure of rDNA which demonstrated that the PGRA is able to detect restriction fragments corresponding to transcriptionally active genes as long as they are located in non-pericentric chromosomal sites. The high density of silencing complexes within pericentric regions is likely to restrict drastically the proportion of repeats devoid of canonical nucleosomes. Repressive complex assembly requires, among other components, methylation of H3K9 [22] that is prominent in pericentric heterochromatin of all organisms that have been studied to date. Within such a context, questions that remain are how transcriptionally active rRNA genes are selected and how they escape from inactivation.

## Materials and Methods

### Animals

Fourth instar larvae of *Camptochironomus tentans* (formerly named *Chironomus tentans*; Diptera: Chironomidae*), Chironomus riparius* (formerly named *Chironomus thummi* and *Sciara coprophila* (Diptera: Sciaridae) came from laboratory stocks at the CIB-CSIC (Madrid). *Rhynchosciara americana* larvae were collected in the coastal region of Mongaguá, state of São Paulo, Brazil. No specific permits were required for collecting this species as only locations that are not protected were visited to this end. When *Rhynchosciara* was occasionally collected within coastal private land, formal permission was not necessary as it was provided orally by the owner at the collection day. Also, *Rhynchosciara* is not considered endangered or protected species. *Drosophila melanogaster* (Canton S) and *Trichomegalosphys pubescens* (Diptera: Sciaridae; formerly named *Trichosia pubescens*) came from laboratory stocks at the IB-USP, São Paulo. The rDNA map of the latter species was constructed based on Southern-blot hybridization results of genomic DNA cut with several restriction enzymes probed with labeled rDNA fragments specific for 18S and 28S rRNA genes. The probes came from selected restriction fragments of cloned rDNAs of *R. americana* [49], *C. riparius* [47] and *D. melanogaste*r [18].

### Psoralen Photocrosslinking

Salivary glands ovaries and testes were dissected and placed in an open plate containing 0.3 ml TE buffer (10 mM Tris-HCl, 1 mM EDTA, pH 7.6). The plate was kept on ice until the end of the procedure. Irradiation was performed in the presence of 4,5,8-trimethylpsoralen (Trioxalen, Sigma) using a 365 nm UV lamp (UVP B-100A or Spectronics SB-100F) at a distance of 6 cm as previously described [13]. Psoralen solution (0.2 mg/ml) was added (0.05V) six times to the TE buffer at intervals of 5 min, for a total irradiation time of 30 min. Solutions containing DNA size markers or plasmid constructions cut with restriction enzymes were irradiated as described above except for the total irradiation time that varied from 5 min to 30 min.

### DNA Extraction and Gel Electrophoresis

After recovering psoralen-treated tissues by centrifugation, the samples were resuspended in lysis buffer (15 mM NaCl, 0.1 mM EDTA, 15 mM Tris-HCl pH 8, 0.5% SDS, 0.2 mg/ml proteinase-K) and incubated at 60°C overnight. After one phenol/chlorophorm extraction the DNA solution was treated for 1 h at 37°C with RNase A. After two phenol/chloroform extractions the DNA was precipitated with sodium acetate 3 M, pH 5.2 (0.1V) and ethanol (2.5V) at −20°C overnight. After centrifugation the supernatant was discarded and the pellet resuspended in water. For genomic DNA extraction without psoralen treatment, larvae were frozen on dry ice, homogenized in TES buffer (100 mM NaCl, 10 mM Tris-HCl pH 8, 25 mM EDTA, 0.5% SDS, 0.2 mg/ml proteinase-K) following procedures described above. The electrophoretic separation of DNA fragments from the chromatin was performed in 1.2% agarose gels at 3 V/cm for 17 h with buffer re-circulation. Before blotting, both sides of the gels were irradiated with short-wavelength UV light (150 mJ/side, GS Genelinker, Bio-Rad) to reverse the psoralen crosslinking. Restriction enzymes and DNA size markers were purchased from Roche and Fermentas, respectively.

### Transfer, Hybridization and Quantification

Alkaline transfer of DNA to membranes was made according to standard protocols (Bio-Rad or GE Healthcare). Probes from eluted plasmid inserts were labeled with α^32^P-dCTP by random priming with either Ready-To-Go DNA labeling beads (GE Healthcare) or Random Primers DNA Labeling System (Invitrogen). Removal of unincorporated nucleotides was done using ProbeQuant G-50 micro-columns (GE Healthcare). Hybridization was carried out overnight at 60°C in 0.5 M Na_2_HPO_4_, pH 7.2, 4% SDS. Washes were done twice at 60°C for 20 min in 40 mM Na_2_HPO_4_, pH 7.2, 4% SDS. Agfa X-ray films were exposed without amplifier screens. Quantification was performed with Phosphorimager (Molecular Dynamics) and ImageQuant software. Image processing of Fig. 6B was performed with Adobe Photoshop 5.0.

### Preparation of Chromosome Spreads

The following fixation mixture was used: 2% formaldehyde (Merck), 7 mM K_2_HPO_4_, 3 mM KH_2_PO_4_, 100 mM NaCl, 2 mM KCl, 2% NP-40. The fixed tissues were then squashed in 50% acetic acid/formaldehyde (3.7%) and frozen in liquid nitrogen. The coverslips were pried off with a razor blade and the slides were kept in absolute ethanol at –20°C until the immunological detection.

### Immunostaining

The slides were rehydrated in 1X TBS followed by incubation at room temperature in 1X TBS, 0.1% Triton X-100 (TBST), for 20–30 min. Detection of trimethyl-histone H3 at lysine 9 was carried out with rabbit IgG anti-trimethyl H3K9 (07–442, Upstate) diluted 1∶50 in TBST. The incubations were done in a moistened chamber at room temperature for 2 h. After washes in TBST, the slides were incubated for 1 h at room temperature with sheep IgG anti-rabbit conjugated with TRITC (Sigma) diluted 1∶100 in TBST solution. The slides were washed twice in TBST for 30 min and finally in 1X TBS for 5 min. The slides were mounted in antifading medium (Vectashield, Vector Labs) and inspected with epifluorescence optics (Axiophot II, Zeiss) microscope equipped with a CCD camera (PCO).
